# Assessing the Resilience of Machine Learning Classification Algorithms on SARS-CoV-2 Genome Sequences Generated with Long-Read Specific Errors

**DOI:** 10.3390/biom13060934

**Published:** 2023-06-02

**Authors:** Bikram Sahoo, Sarwan Ali, Pin-Yu Chen, Murray Patterson, Alexander Zelikovsky

**Affiliations:** 1 Department of Computer Science, Georgia State University, Atlanta, GA 30303, USA; sali85@gsu.edu (S.A.); mpatterson30@gsu.edu (M.P.); 2 IBM Research, IBM T. J. Watson Research Center, Yorktown Heights, NY 10598, USA; pin-yu.chen@ibm.com

**Keywords:** sequencing error, third-generation single-molecule sequencing (TGS), long read, machine learning, embedding methods, classification

## Abstract

The emergence of third-generation single-molecule sequencing (TGS) technology has revolutionized the generation of long reads, which are essential for genome assembly and have been widely employed in sequencing the SARS-CoV-2 virus during the COVID-19 pandemic. Although long-read sequencing has been crucial in understanding the evolution and transmission of the virus, the high error rate associated with these reads can lead to inadequate genome assembly and downstream biological interpretation. In this study, we evaluate the accuracy and robustness of machine learning (ML) models using six different embedding techniques on SARS-CoV-2 error-incorporated genome sequences. Our analysis includes two types of error-incorporated genome sequences: those generated using simulation tools to emulate error profiles of long-read sequencing platforms and those generated by introducing random errors. We show that the spaced *k*-mers embedding method achieves high accuracy in classifying error-free SARS-CoV-2 genome sequences, and the spaced *k*-mers and weighted *k*-mers embedding methods are highly accurate in predicting error-incorporated sequences. The fixed-length vectors generated by these methods contribute to the high accuracy achieved. Our study provides valuable insights for researchers to effectively evaluate ML models and gain a better understanding of the approach for accurate identification of critical SARS-CoV-2 genome sequences.

## 1. Introduction

During the COVID-19 pandemic, whole genome sequencing (WGS) of the SARS-CoV-2 virus has played a crucial role in unraveling important biological information. Through phylogenetic analysis, it has been revealed that SARS-CoV-2 shares 50% and 79% sequence similarity with MERS-CoV and SARS-CoV, respectively, indicating their evolutionary connections [1]. Notably, the genome sequence of SARS-CoV-2 exhibits an 85% similarity to a bat coronavirus, establishing its zoonotic origin within the Coronaviridae family and the Betacoronavirus genus [2]. These genomic data have been instrumental in confirming the virus’s source and classification. Recognizing the significance of gathering genetic data from diverse SARS-CoV-2 sequences and variants, researchers worldwide swiftly recognized the need for comprehensive genome information [3,4]. The Centers for Disease Control and Prevention’s Office of Advanced Molecular Detection (AMD) released details regarding SARS-CoV-2 whole genome sequencing on various platforms, including PacBio, Illumina, and Ion Torrent. Emphasizing the importance of publicly accessible genome sequences, the World Health Organization (WHO) strongly supports their utilization in developing novel public health strategies and conducting research to combat the spread of COVID-19. A valuable resource in this endeavor is the Global Initiative on Sharing All Influenza Data (GISAID), which hosts one of the largest international databases of SARS-CoV-2 genome sequences [5]. Leveraging GISAID, along with the open-source tools NextStrain and NextClade, researchers have made significant advancements in their investigations [6,7]. These resources have proven instrumental in understanding the evolution and characteristics of the virus, aiding in the development of efficient strategies to mitigate the COVID-19 infection’s spread [8,9,10].

Third-generation sequencing technology has emerged as a widely used method for sequencing SARS-CoV-2 during the pandemic. These technologies, known for their ability to generate long reads, are increasingly employed in transcriptomics studies. Advancements in long-read sequencing enable the comprehensive sequencing of RNA molecules, utilizing cDNA or direct RNA protocols from Oxford Nanopore Technologies (ONT) and Pacific Biosciences (PacBio) [11,12,13]. However, the high error rates associated with long-read technologies pose challenges for accurate and efficient downstream analysis, such as genome assembly. Indels, or insertions and deletions, are the primary error types that complicate alignment processes. While various error correction tools exist, there remains a need for further development in this computational biology domain. To effectively combat the COVID-19 infection and facilitate research, an increased number of SARS-CoV-2 genome sequences are required [14,15]. Researchers worldwide rely on third-generation sequencing technologies to sequence the virus. Cutting-edge technology heavily relies on SARS-CoV-2 genomic sequences for virus tracking. To analyze genomic data effectively, scientists employ machine learning (ML) and deep learning (DL) algorithms along with embedding methods for classification purposes [16,17,18,19]. ML and DL algorithms have become valuable tools even for novice bioinformatics practitioners and core data analysts who may lack prior knowledge of sequencing technologies and associated challenges. These algorithms enable comprehensive analysis of SARS-CoV-2 sequencing data, contributing to advancements in classification techniques and aiding in our understanding of the virus’s genetic characteristics and behavior. Therefore, it is crucial to establish a robust benchmark report on SARS-CoV-2 genome sequences generated using third-generation sequencing technology, which will serve as a guide for future genomic research involving long-read sequencing.

**Figure 1:**
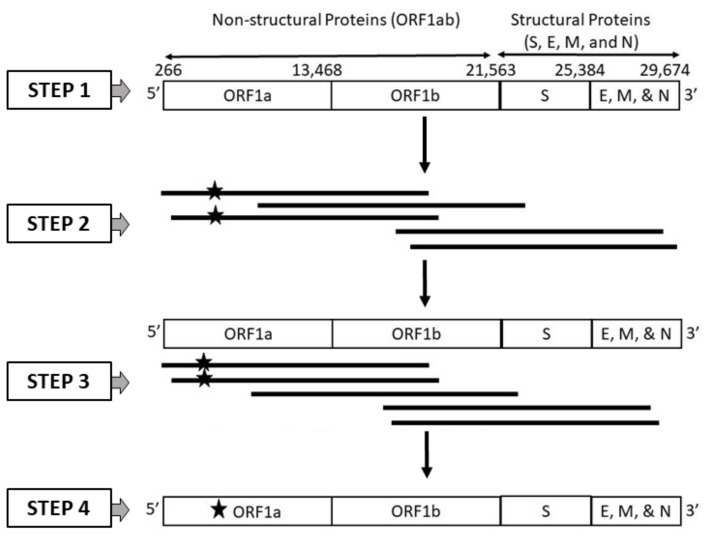
Workflow employed for generating a dataset incorporating long-read (PacBio and ONT) specific errors, which was subsequently used to evaluate the robustness of machine learning models employing different embedding techniques. The workflow comprises four distinct steps: 1. collection of high-quality SARS-CoV-2 reference genome sequences from GISAID, 2. generating long-read sequences tailored to PacBio and ONT sequencers, incorporating sequencer-specific errors (represented as star), utilizing the reference genome for the ORF1a gene, 3. aligning the error-incorporated long reads (reads marked with star) to the reference genome, and 4. creating the final compilation of SARS-CoV-2 genome sequences, encompassing long-read sequencer-specific errors (represented as star) in the ORF1a gene.

The current study aims to evaluate the performance of current classification models in handling third-generation sequencer-specific errors present in SARS-CoV-2 genome sequences. Specifically, the study investigates the effectiveness of various embedding methods under specified levels of disturbance. The evaluation of machine learning models on SARS-CoV-2 genomic sequences remains limited, with only a few existing studies in this area. For instance, a previous study [20] conducted a benchmark of ML and DL models using different embedding methods for classifying SARS-CoV-2 genome sequences that included sequencer-specific errors. However, this study did not identify the best ML model for SARS-CoV-2 genome sequence classification. In line with a similar approach, our current study focuses exclusively on SARS-CoV-2 genomes generated using long reads obtained from third-generation sequencing (TGS) technologies, such as PacBio and Nanopore, while also considering the possibility of random errors occurring by chance. To assess the effectiveness of machine learning algorithms on SARS-CoV-2 genome sequences, we conducted simulations that accounted for various error types. Our simulations employed two primary approaches: one involved generating SARS-CoV-2 genome sequences with platform-specific errors (PacBio or ONT), while the other introduced random errors. The workflow for these simulations is depicted in Figure 1. To analyze the SARS-CoV-2 sequences, we employed six distinct embedding methods, including one-hot encoding (OHE), Wasserstein-distance-guided representation learning (WDGRL), string kernel, spaced *k* -mers, weighted *k*-mers, and weighted position weight matrix (PWM). Leveraging these embedding methods, we performed supervised analyses using a variety of linear and non-linear classifiers considering both clean and error-incorporated SARS-CoV-2 sequences. This comprehensive methodology enabled us to evaluate the effectiveness of these methods in detecting errors and classifying sequences.

The subsequent sections of the current study are described in an arranged manner as follows. Section 2 comprises comprehensive details of the dataset statistics, dataset generation methodology, and various embedding techniques considered to convert SARS-CoV-2 genome sequences to fixed-length numerical representations. Our results for accuracy and robustness are reported in Section 3. Finally, the current study concludes in Section 4.

## 2. Dataset and Methodology

This section is devoted to the elucidation of the datasets utilized in this study and the process through which the validation dataset incorporating long-read specific error models (PacBio and ONT) and random errors was generated (refer to Section 2.1). In addition, Section 2.2 provides a succinct overview of the different types of embedding methods employed. The methodology adopted for the development of machine learning classification algorithms and the computation of their accuracy and robustness is presented in Section 2.3. Finally, Section 2.4 expounds on the visualization of the high-dimensional SARS-CoV-2 sequencing data.

### 2.1. Dataset Generation

In this study, four distinct datasets were employed. One of these datasets encompasses all genomes from the Global Initiative on Sharing All Influenza Data (GISAID), which have been meticulously curated to ensure their accuracy. The remaining three datasets were derived from distinct error models. Specifically, two datasets were generated through the use of PacBio and ONT models, while the fourth dataset was produced via a random error model. A detailed exposition of the dataset properties and characteristics is provided in their corresponding sections, namely Section 2.1.1, Section 2.1.2, Section 2.1.3, and Section 2.1.4.

#### 2.1.1. Dataset 1: High-Quality SARS-CoV-2 Genome Sequences

To create a dataset of high-sequencing quality SARS-CoV-2 whole genome sequences, we analyzed 8172 sequences from GISAID between September and December 2021. Our selection criteria focused on complete and high-coverage genome sequences to ensure the collection of high-quality genomes. We specifically limited our sequence collection to those obtained from the human host. Additionally, we gathered lineage information for the sequences, resulting in 41 unique Pango lineages within our dataset. For detailed sequence statistics, please refer to Table 1.

#### 2.1.2. Dataset 2: SARS-CoV-2 Genome Sequences Generated from Long Reads Incorporating PacBio Sequencing Errors

To generate the second SARS-CoV-2 genome sequence dataset, we utilized Pacific Biosciences (PacBio) sequencing technology and simulated long reads with PacBio sequencing errors. This was accomplished using PBSIM, a tool specifically designed to simulate PacBio sequencing reads with varying error rates [21]. PBSIM can generate two types of reads associated with the PacBio sequencer: continuous long reads (CLR) and circular consensus sequencing (CCS) short reads. CCS reads generally exhibit lower error rates compared to CLR reads. PBSIM offers two simulation approaches: sampling-based and model-based simulation, which facilitate the generation of PacBio CCS and CLR reads. In the sampling-based simulation, PBSIM considers the quality and length of the input read set, while the model-based simulation incorporates a built-in error model.

In our study, we employed the model-based approach of PBSIM, utilizing the pbsim-v1.0.3 tool to simulate PacBio long reads with errors based on the genomic sequence of SARS-CoV-2. Subsequently, these erroneous long reads were aligned to the SARS-CoV-2 reference genome (GenBank accession number NC_0455122) using Minimap v2-2.24 [22], and variants were called from the aligned reads using bcftools v1.6 [23]. This process resulted in the generation of a consensus sequence, which represents a SARS-CoV-2 genome sequence incorporating typical long-read errors. We generated simulated reads with errors at two distinct depths, namely 5× and 10×, specifically on Dataset 1, leading to the creation of Dataset 2.

#### 2.1.3. Dataset 3: SARS-CoV-2 Genome Sequences Generated from Long Reads Incorporating Oxford Nanopore Technology (ONT) Sequencing Errors

The third dataset was generated using long reads simulated with an Oxford Nanopore Technologies (ONT) sequencing error profile. To simulate long reads with Nanopore sequencing errors, we employed the Badread software tool, known for incorporating realistic artifacts introduced by Nanopore sequencers, including chimeras, junk reads, glitches, and adapters [24]. Badread utilizes a gamma distribution for read length, allowing for user-specified mean and standard deviation parameters.

We utilized Badread v0.2.0 to simulate ONT (Oxford Nanopore Technologies) long reads with errors based on the genome sequence of SARS-CoV-2. Following this, we aligned these error-prone long reads to the SARS-CoV-2 reference genome (GenBank accession number NC_0455122) using Minimap v2-2.24 [22]. By leveraging bcftools v1.6 [23] to call variants from the aligned reads, we obtained a consensus SARS-CoV-2 sequence that incorporated errors typically associated with Nanopore sequencing technologies. In order to ensure a comprehensive analysis, we generated erroneous simulated reads at two distinct depths: 5× and 10×. These steps were specifically performed on Dataset 1, resulting in the creation of Dataset 3.

#### 2.1.4. Dataset 4: SARS-CoV-2 Genome Sequences Generated from Long Reads Incorporating Random Errors

The fourth dataset of the SARS-CoV-2 genome sequence was generated using long reads with random errors. For this purpose, we utilized the random option in the Badread software tool, known for its ability to simulate long reads with various types of errors, including random errors [24].

By utilizing the random option available in Badread v0.2.0, we simulated long reads with random errors. Subsequently, these long reads were aligned to the SARS-CoV-2 reference genome (GenBank accession number NC_0455122) using Minimap v2-2.24 [22]. We then performed variant calling using bcftools v1.6 [23] and derived a consensus SARS-CoV-2 sequence by incorporating the introduced variants caused by random errors. To ensure a comprehensive analysis, we repeated this procedure at two different read depths, specifically 5× and 10×, which aligns with the approach taken for Datasets 2 and 3. These aforementioned steps were executed on Dataset 1, resulting in the creation of Dataset 4.

### 2.2. Embedding Generation Methods

This section delineates the analytical methodologies used to examine the datasets explicated in the previous section. Six distinct embedding methods, namely one-hot encoding (OHE), Wasserstein-distance-guided representation learning (WDGRL), string kernel, spaced *k*-mers, weighted *k*-mers, and weighted position weight matrix (PWM), were implemented to transform the sequences into machine-readable, low-dimensional numerical embeddings (also known as feature vectors) in this study. The specifics of each method are elaborated upon in their respective sections, namely Section 2.2.1, Section 2.2.2, Section 2.2.3, Section 2.2.4, Section 2.2.5, Section 2.2.6, respectively.

#### 2.2.1. One-Hot Encoding (OHE)

One-hot encoding is a common method for generating numerical embedding from a nucleotide sequence (OHE). OHE represents each nucleotide in the sequence as a binary (0–1) vector; in this case, the nucleotides are A, T, C, and G [17,18]. To illustrate this mathematically for nucleotide sequences, consider a mathematical function (f) that maps each nucleotide to its appropriate one-hot encoding vector. We can write the function as follows:

(1) f(Σ)=(One-hotencodedvector)

Suppose in Equation (1), Σ is a one of the nucleotides from {A,T,C,G}. For instance,

$$\begin{array}{c} f(A)=(1,0,0,0)\\ f(C)=(0,1,0,0)\\ f(G)=(0,0,1,0)\\ f(T)=(0,0,0,1) \end{array}$$

After this, we can concatenate all the one-hot encoded vectors generated for individual nucleotides using function (f) from a DNA sequence to get a conclusive embedding vector.

Using the above concept, suppose a DNA sequence (X) of length (n) can then be represented by creating a final binary vector ($\phi_{x}$) in Equation (2) by concatenating all the individual components (X) generated using the function (f).

(2) $$\phi_{x}=\left(f\left(X_{1}\right),f\left(X_{2}\right),\ldots ,f\left(X_{n}\right)\right)$$

In the above equation, $X_{i}$ is the nucleotide in DNA sequence X at position i. The $\phi_{x}$’s dimension in this instance is |Σ|× n (|Σ| is the size of the nucleotide alphabet, i.e., 4 in this case.)

#### 2.2.2. Wasserstein-Distance-Based Generative Adversarial Network for Representation Learning (WDGRL)

The Wasserstein-distance-based generative adversarial network for representation learning (WDGRL) is an unsupervised method intended to generate a low-dimensional embedding [25]. It accomplishes the goal by extracting the features from input data using a neural network-based model that takes advantage of the source and encoded target data distribution. The model determines the Wasserstein distance (WD) between the original high-dimensional vector and low-dimensional representation. WDGRL considers one-hot encoded (OHE) vectors as input and generates a very-low-dimensional representation that consists solely of essential features.

Let us consider *X* numbers of one-hot encoded (OHE) SARS-COV-2 genome sequences as input data *D* to the neural network model $M_{\theta }$. The model $M_{\theta }$ with parameters θ maps every OHE vector $X_{i}$ from *X* to a low-dimensional representation $h_{i}$ in $R^{d}$. During this process, $M_{\theta }$ learns to generate an $h_{i}$ that captures the essential features from $X_{i}$ by considering the encoded distribution of the source and the target data.

Suppose the distributions of the encoded representation of $h_{i}$ for the source and target data are $P_{s}\left(h\right)$ and $P_{t}\left(h\right)$. Here, $P_{s}\left(h\right)$ and $P_{t}\left(h\right)$ can be estimated by a density estimation method. The loss function for the WDGRL can be written as

(3) $$L\left(\theta \right)=maxf(E\left[h_{s}P_{s}\left[f\left(h_{s}\right)\right]]-E\left[h_{t}P_{t}\left[f\left(h_{t}\right)\right]\right]\right)$$

In Equation (3), *f* is a Lipschitz-1 function to approximate the Wasserstein distance between the distributions $P_{s}\left(h\right)$ and $P_{t}\left(h\right)$. Here, the function f(h) can be parameterized by another neural network $D_{\alpha }$, which considers the encoded representation of $h_{i}$ as input and outputs a scalar value. The loss function is optimized by minimizing the negative of its value with respect to the parameter’s θ and α:

(4) $$L\left(\theta ,\alpha \right)=-L\left(\theta \right)=\min_{\theta ,\alpha }(E\left[h_{s}P_{s}\left[D_{\alpha }\left(h_{s}\right)\right]]-E\left[h_{t}P_{t}\left[D_{\alpha }\left(h_{t}\right)\right]\right]\right)$$

The neural networks $G_{\theta }$ and $D_{\alpha }$ are jointly trained to minimize the WDGRL loss function using gradient descent or other optimization methods. The resulting low-dimensional representation captures important features of the input data that are useful for downstream tasks, such as classification.

#### 2.2.3. String Kernel

The string kernel method operates in the non-Euclidean space and measures the similarity between the SARS-CoV-2 genome sequences by computing a kernel matrix, also known as a Gram matrix [26].

Given a set of SARS-COV-2 genome sequences $X=\{X_{1},X_{2},X_{3},\ldots .,X_{n}\}$, the string kernel method first computes the *k*-mers of length k; for the current scenario, k is 3 for each genome sequence. The term *k*-mer refers to a substring of length k that occurs in the SARS-CoV-2 genome sequence.

Let us consider M be the matrix of all possible *k*-mers for a SARS-CoV-2 genome sequence *j*, and $M_{ij}$ is the number of times the *k*-mer *i* occurs in the sequence *j*. Then, the similarity between two SAR-CoV-2 genome sequences $X_{i}$ and $X_{j}$ can be computed using the kernel function, Equation (5).

(5) $$K\left(x_{i},x_{j}\right)=\sum_{i\in x_{i}}\sum_{j\in x_{j}}M_{ik}*M_{jk}$$

Here, $M_{ik}$ and $M_{jk}$ are the number of occurrences of k-mer *i* and *j* in sequences $X_{i}$ and $X_{j}$, respectively.

To reduce the computational complexity, the string kernel method considers a locally sensitive hashing-based approach to estimate the *k*-mers of two sequences at distance m from each other. This approach hashes *k*-mers into bins considering their locality and further uses the bin information to estimate the matching *k*-mers between the two SARS-CoV-2 sequences. The resulting kernel matrix K from Equation (5) is a symmetric matrix, and $K_{ij}$ is the kernel value between the genome sequences $X_{i}$ and $X_{j}$.

To generate a low-dimensional representation of the input genome sequences, we performed kernel principal component analysis (PCA) on the kernel matrix K. Kernel PCA is a nonlinear dimensionality reduction technique that maps the input data to a new space defined by the eigenvectors of the kernel matrix. The top components are further considered as the reduced dimensional feature vector for the downstream analysis.

Suppose for the kernel matrix K, V is the matrix of eigenvectors and Lambda is the diagonal matrix of the corresponding eigenvectors. Then, the top k principal components are represented by:

(6) $${\mathrm{PC}}_{k}={\left(\lambda_{k}\right)}^{1/2}V\left[:,k\right]$$

where V[:,k] is the k-th column of the matrix V and $\lambda_{k}$ is the k-th diagonal element of Lambda. For this analysis, the top 500 principal components are selected by considering a standard validation approach, and these components are used as the final feature vector for each SARS-CoV-2 genome sequence for downstream tasks such as classification.

#### 2.2.4. Spaced *k*-Mers

The spaced *k*-mers method is used to reduce the sparsity and size of *k*-mers (nucleotide substrings of length k) in the SARS-CoV-2 genome sequence [19]. Given a SARS-CoV-2 genome sequence S, the spaced *k*-mers method first computes *g*-mers. Here, *g*-mers are nucleotide subsequences of length g (where g is an integer > 1). From those *g*-mers, the method then computes *k*-mers, where k < g. While generating *k*-mers from *g*-mers, the method skips some of the characters (nucleotides) between adjacent *g*-mers. The size of the gap between adjacent *g*-mers is determined by g-k.

Formally, suppose S is a SARS-CoV-2 genome sequence of length L. Then, the definition of *g*-mers is as follows:

(7) $$G=\{S_{i}:i+g-1|i=1,2,\ldots ,L-g+1\}$$

(Here, $S_{i}$: *i* + *g* − 1 represents the genomic subsequences of S starting at position i and ending at position i + g − 1.)

From the above computed set of G mers, the method computes the set of *k*-mers as follows:

(8) $$K=\{S_{i}:i+k-1|i=1,2,\ldots ,g-k+1\}$$

(Here, $S_{i}$: *i* + *k* − 1 represents the genomic subsequences of S starting at position i and ending at position i + k − 1.)

After computing the k-mers, the method generates a numerical vector of length ${\left|\Sigma \right|}^{k}$ (where Σ corresponds to the alphabet A, C, G, T).

In our case, the spaced *k*-mer method considers k = 4 and g = 9, which is determined using a standard validation approach. This means we compute *k*-mers of length 4 from SARS-CoV-2 genomic subsequences of length 9, with a gap of 5 nucleotide between adjacent subsequences.

#### 2.2.5. Weighted *k*-Mers

The weighted *k*-mers-based spectrum method is used to denote biological sequences as fixed-length vectors that capture the occurrences of all possible *k*-mers (k represents the length of the subsequences). The method allocates weights to the *k*-mers that are calculated based on their inverse document frequency (IDF), which determines how uncommon a particular *k*-mer is across all sequences.

Formally, to compute the IDF weights, we first calculate the total number of input SARS-CoV-2 genome sequences, N, and the number of input genome sequences that contain a specific *k*-mer, $n_{i}$. The IDF weight for the *k*-mer i is then provided by:

(9) $$\mathrm{IDF}\left(i\right)=\log\left(N/n_{i}\right)$$

Then, we generate a list of all possible *k*-mers based on the nucleotide set A, C, G, T of the input SARS-CoV-2 genome sequences. For each SARS-CoV-2 genome sequence, we calculate the frequency of each *k*-mer and multiply it by the corresponding IDF weight to obtain a weighted frequency, which is given by:

(10) w(i,j)=f(i,j)*IDF(i)

Here, w(i,j) is the weighted frequency of *k*-mer i in SARS-CoV-2 genome sequence j, f(i,j) is the frequency of *k*-mer i in SARS-CoV-2 genome sequence j, and IDF(i) is the IDF weight of *k*-mer i.

The above weighted frequency values are then used to construct a frequency vector, where each element represents the frequency of a particular *k*-mer in the sequence. The frequency vector for sequence j is given by:

(11) v(j)=[w(1,j),w(2,j),…,w(m,j)]

where m is the total number of possible *k*-mers. In our experiment, we considered k = 3, which is selected by a standard validation approach.

#### 2.2.6. The Weighted Position Weight Matrix (PWM)

The weighted position weight matrix (PWM) technique generates PWM scores for all possible *k*-mers (k is the length of subsequences) in SARS-CoV-2 genome sequences [27]. The PWM is produced by a two-step method: In the first step, the method calculates the occurrence of each base at every position of the *k*-mers. In the second step, it computes a weighted score for each *k*-mer considering the log-odd ratio of its observed frequency compared to the background frequency. The background frequency is estimated using the LaPlace pseudocount and the equal probability assumption for each nucleotide.

The formula for computing the PWM is as follows:

(12) $$\mathrm{PWM}\left(k-\mathrm{mer}\right)=\sum_{i=1}^{K}\log_{2}\left(\frac{f(k{-\mathrm{mer}}_{i},b)}{b_{i}}\right)$$

where *k*-mer is the *k*-mer sequence, K is the length of the *k*-mer, f(k−meri,b) is the count of the nucleotide *b* at position i in the *k* − mer, $b_{i}$ is the background frequency of the nucleotide b, and log2 is the base-2 logarithm function.

The final output is a list of scores for each k-mer in each input sequence, where the score is the sum of the weight scores for each base in the k-mer. For the current experiment, k = 3 was selected using the standard validation set approach.

### 2.3. Machine Learning Classification Algorithms: SVM, NB, MLP, KNN, RF, LR, and DT

To perform the classification task, we utilize seven machine learning algorithms: Support Vector Machine (SVM), Naïve Bayes (NB), Multi-Layer Perceptron (MLP), K-Nearest Neighbors (KNN), Random Forest (RF), Logistic Regression (LR), and Decision Tree (DT). Our objective is to evaluate the performance of these algorithms by employing two different approaches.

#### 2.3.1. Approach 1: Accuracy

Here, we compute the average accuracy, precision, recall, F1 (weighted), F1 (Macro), and ROC-AUC for the entire dataset, including all the class labels mentioned in Table 1. We exclude error sequences from the dataset.

#### 2.3.2. Approach 2: Robustness

Robustness is crucial to machine learning models. It represents their ability to generate reasonable outputs for input examples not included in the training data. As our test set, we consider only PacBio, ONT, and random protocol-specific errors incorporating noisy examples, whereas the training set uses non-errored sequences. We then calculate the average accuracy, precision, recall, F1 (weighted), F1 (Macro), and ROC-AUC for the ML models based on the test set. Overall, these two strategies provide comprehensive evaluations of machine learning algorithms’ performance. This allows us to compare and identify the most-suitable algorithm for our classification task.

### 2.4. Data Visualization

In order to ascertain if there exists any inherent clustering in our dataset, we employ the t-distributed stochastic neighbor embedding (t-SNE) approach to produce a two-dimensional representation of the feature embeddings [28].

## 3. Results and Discussion

This section provides an overview of the outcomes achieved by our methods on the datasets employed in this study. The first subsection, labeled Section 3.1, discusses the accuracy evaluation of machine learning classification algorithms that utilized various embedding methods. The second subsection, labeled Section 3.2, covers the robustness evaluation of machine learning classification algorithms that used different embedding methods. The third subsection, labeled Section 3.3, focuses on the comparison of predictive performance of machine learning models on SARS-CoV-2 sequences with errors obtained from PacBio and ONT sequencers. Lastly, Section 3.4 explores the analysis of coronavirus variants using various embedding vector generation methods with the aid of t-SNE visualization.

### 3.1. Accuracy Evaluation of Machine Learning Classification Algorithms Using Different Embedding Methods

**Figure 2:**
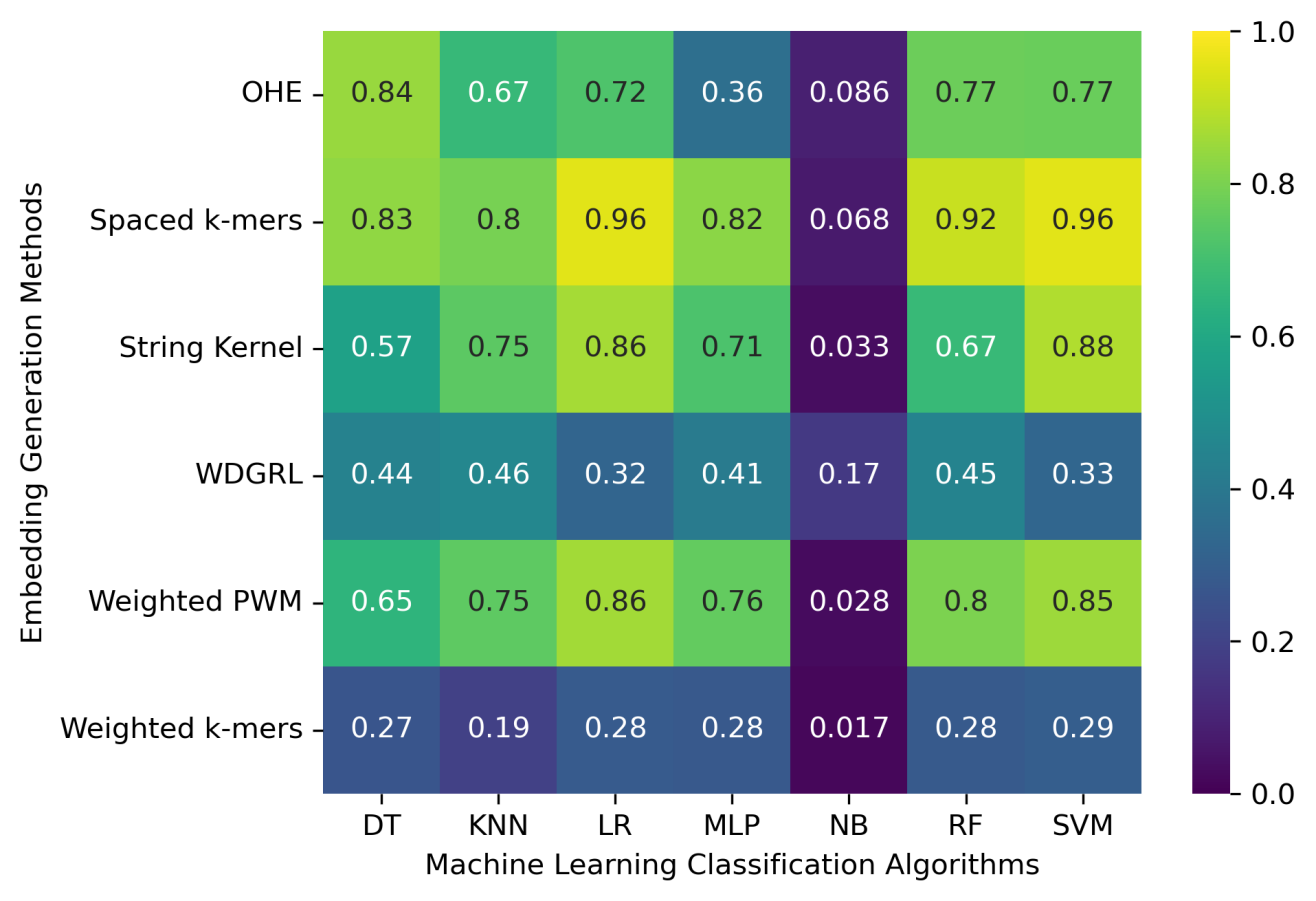
Represents the accuracies of machine learning classification algorithms obtained from an error-free set of 8172 SARS-CoV-2 genome sequences with different embedding generation methods.

We considered 8172 clean (error-free) full-length SARS-CoV-2 nucleotide sequences from the GISAID database. These sequences were used to evaluate the machine learning models with embedding methods. In order to do that, we split the sequences into training and test sets with a 70/30% ratio. After that, we executed each analysis five times and considered the average results, reported in Table 2 and Figure 2. The results show that the machine learning classification algorithms’ performance significantly varies depending on the embedding method employed. Specifically, the one-hot embedding method leads to an accuracy of 0.773 for the SVM algorithm, whereas the WDGRL embedding method only results in an accuracy of 0.327. The spaced *k*-mers embedding method with the SVM, RF, LR, and DT classification algorithms achieves an accuracy of up to 0.956. This method employs *g*-mers and *k*-mers to decrease the sparsity and size of *k*-mers in the genome sequence. As a result, it generates fixed-length vectors that capture the occurrences of all possible *k*-mers, which are then used to construct frequency vectors representing the frequency of each *k*-mer in the sequence. This method performs well with the error-free set of SARS-CoV-2 genome sequences. However, the NB algorithm yields the worst results, with an accuracy of only 0.017 when the weighted *k*-mers embedding method is used. Additionally, some algorithms, such as SVM and LR, have significantly longer training times compared to others. Thus, while selecting an algorithm and embedding method, one should consider both performance and training time.

### 3.2. Robustness Evaluation of Machine Learning Classification Algorithms Using Different Embedding Methods

We considered 8172 clean SARS-CoV-2 sequences and incorporated errors specific to PacBio, ONT, and the random protocol, as described in the methods section. This approach helped to generate three different types of datasets: genome sequences with typical PacBio sequencing errors, ONT sequencing errors, and random errors. To evaluate the robustness of the machine learning models with embedding methods on the three different datasets, we train the models with clean SARS-CoV-2 sequences and test them on error-incorporated sequences.

#### 3.2.1. The Robustness Results for PacBio Sequencing Error-Incorporated Datasets

**Figure 3:**
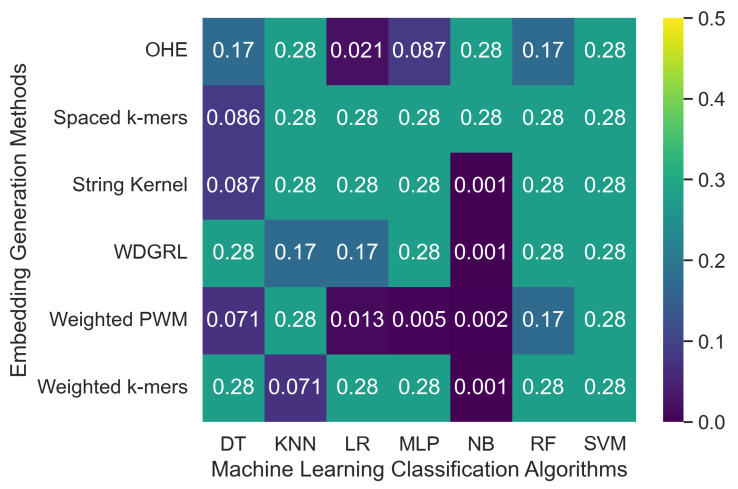

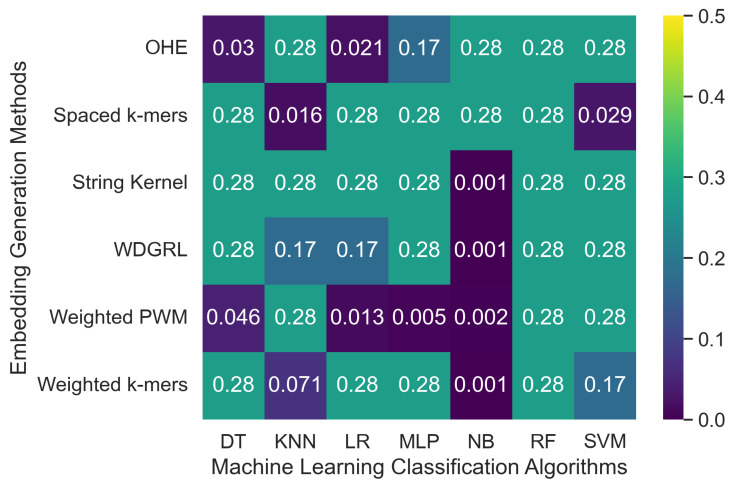
Represents the robustness of machine learning classification algorithms with respect to specific errors related to the PacBio sequencer on a set of 8172 SARS-CoV-2 genome sequences. The analysis was carried out using various embedding generation techniques, and the results were obtained separately for two different sequencing depths: 5 and 10. The presentation of results is segregated into two sections, with the top section representing the outcomes for a depth of 5 and the bottom section representing the findings for a depth of 10.

Table 3 displays the accuracy values of various machine learning classification algorithms that used different embedding methods on SARS-CoV-2 genome sequence datasets simulated at two different depths, 5 and 10, with PacBio sequencer-specific errors incorporated. Furthermore, Figure 3 reveals that the accuracy values for machine learning algorithms ranged from 0.001 to 0.276 across all embedding methods. The spaced *k*-mers embedding method, in general, performed better than other embedding methods, achieving the highest accuracy value of 0.276 for the maximum number of algorithms for the depth-5 sequencing dataset, and a similar trend was observed for the depth-10 dataset. The reason for this is that the spaced *k*-mers method employs *g*-mers and *k*-mers to decrease the sparsity and size of *k*-mers in the genome sequence. As a result, it generates fixed-length vectors that capture the occurrences of all possible *k*-mers, which are then used to construct frequency vectors representing the frequency of each *k*-mer in the sequence. The accuracy results confirm that as the depth decreases, the error rate increases, resulting in a decrease in the performance of machine learning models. The model’s performance did not improve significantly by increasing sequencing depth from 5 between the two SARS-CoV-2 genome sequence datasets.

#### 3.2.2. The Robustness Results for Oxford Nanopore Technologies (ONT) Sequencing Error-Incorporated Datasets

**Figure 4:**
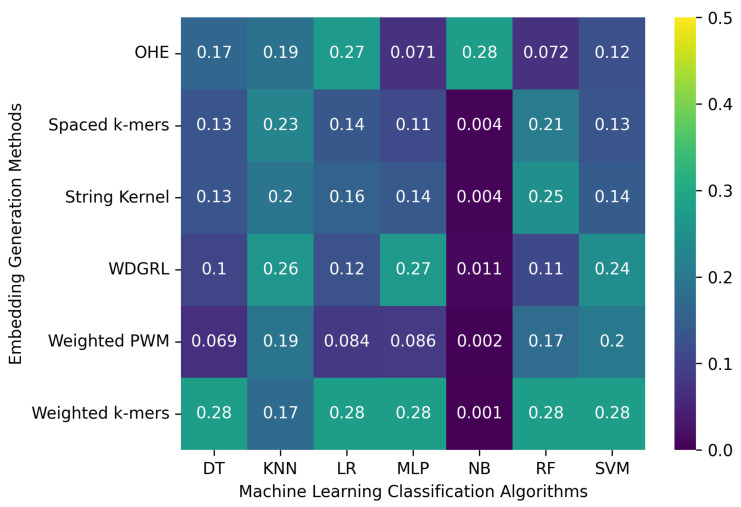

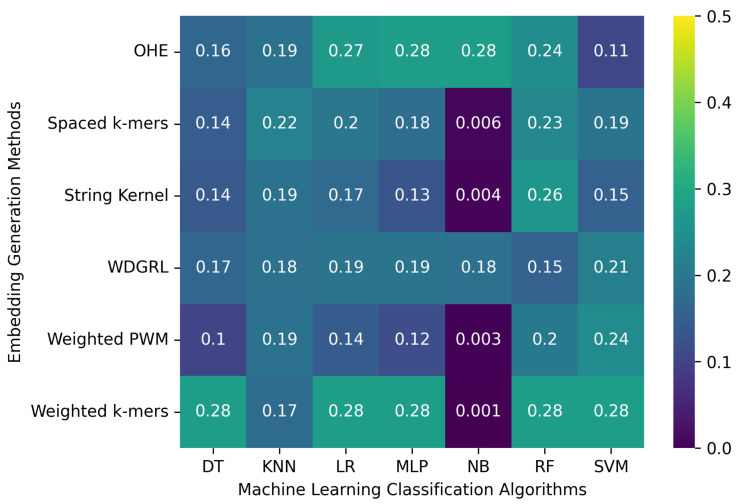
Represents the robustness of machine learning classification algorithms with respect to specific errors related to the Oxford Nanopore sequencer on a set of 8172 SARS-CoV-2 genome sequences. The analysis was carried out using various embedding generation techniques, and the results were obtained separately for two different sequencing depths: 5 and 10. The presentation of results is segregated into two sections, with the top section representing the outcomes for a depth of 5 and the bottom section representing the findings for a depth of 10.

Table 4 displays the accuracy values obtained from different machine learning algorithms using various embedding methods on two SARS-CoV-2 genome sequence datasets with depths of 5 and 10, respectively, which were generated from long-reads containing Oxford Nanopore Technology (ONT) sequencer-specific errors. Moreover, Figure 4 presents a heatmap that visualizes the accuracy values, which ranged from 0.001 to 0.276. The weighted *k*-mers embedding method resulted in the highest accuracy values for the majority of the machine learning algorithms on both datasets, i.e., depths of 5 and 10. Because each *k*-mer is given a weight depending on its inverse document frequency under the weighted *k*-mers technique, this method generates fixed-length vectors that capture the existence of all potential *k*-mers. These vectors are then used to create frequency vectors that indicate the frequency of each *k*-mer in the sequence. However, due to the lower sequencing depth with ONT sequencer-specific errors, poor-quality SARS-CoV-2 genome sequences were generated, leading to a significant decrease in the predictive performance of machine learning algorithms.

#### 3.2.3. The Robustness Results for Random-Error-Incorporated Datasets

**Figure 5:**
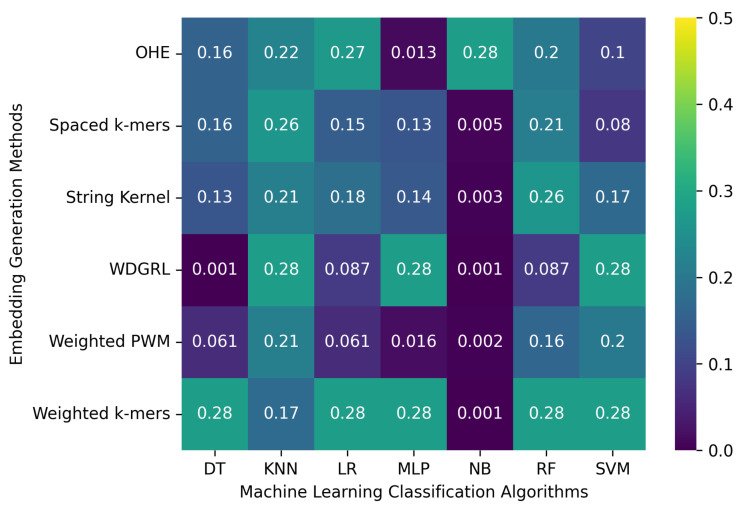

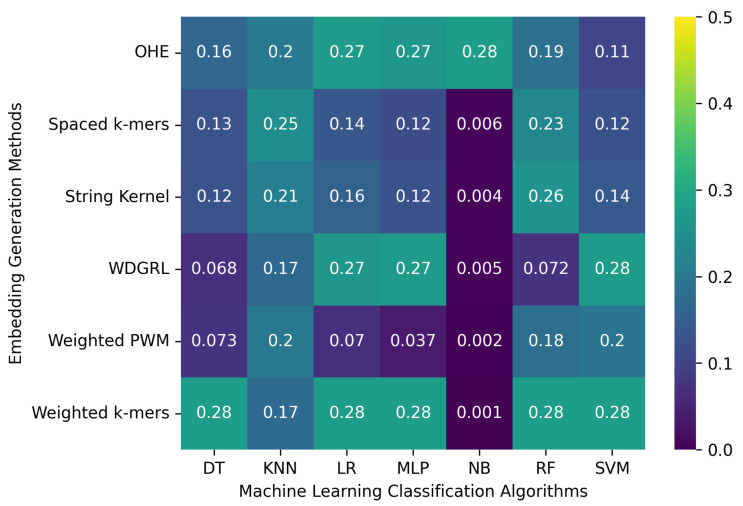
Represents the robustness of machine learning classification algorithms with respect to random errors incorporated into a set of 8172 SARS-CoV-2 genome sequences. The analysis was carried out using various embedding generation techniques, and the results were obtained separately for two different sequencing depths: 5 and 10. The presentation of results is segregated into two sections, with the top section representing the outcomes for a depth of 5 and the bottom section representing the findings for a depth of 10.

In this section, we evaluated the accuracy of various machine learning algorithms using different embedding methods on two SARS-CoV-2 genome sequence datasets. These datasets were generated by incorporating random errors into long-reads at depths of 5 and 10. The results, presented in Table 5 and Figure 5, indicate that the weighted *k*-mers method achieved the highest accuracy of 0.276 across the majority of machine learning classification algorithms for both datasets. The main objective of incorporating random errors into the SARS-CoV-2 datasets was to compare the performance of machine learning models on datasets generated by different types of errors, including sequencer-specific errors and random errors. Interestingly, we found that there was not much difference in accuracy between these two types of errors.

### 3.3. Comparison of Predictive Performance of Machine Learning Models on SARS-CoV-2 Sequences with Errors from PacBio and ONT Sequencers

Third-generation sequencing (TGS) technologies such as PacBio and Oxford Nanopore Technology (ONT) are widely used for generating long reads with high error rates. However, PacBio technology sequences a DNA molecule multiple times, whereas ONT sequences it only twice, making PacBio generate higher-quality data with lower error rates compared to ONT. Through our analysis, we discovered that the errors specific to the PacBio sequencer have a more significant impact on the predictive performance of machine learning (ML) models on SARS-CoV-2 sequences than errors specific to ONT. Our ML model’s predictive performance indicated that PacBio sequences have a lower error rate than ONT, but the low predictive power was due to low coverage. We also compared the predictive performance of ML models on SARS-CoV-2 sequences incorporated with random errors with other datasets and observed that the results were similar to the ONT scenario.

### 3.4. Analysis of Coronavirus Variants Based on Different Embedding Vector Generation Methods Using t-SNE Visualization

**Figure 6:**
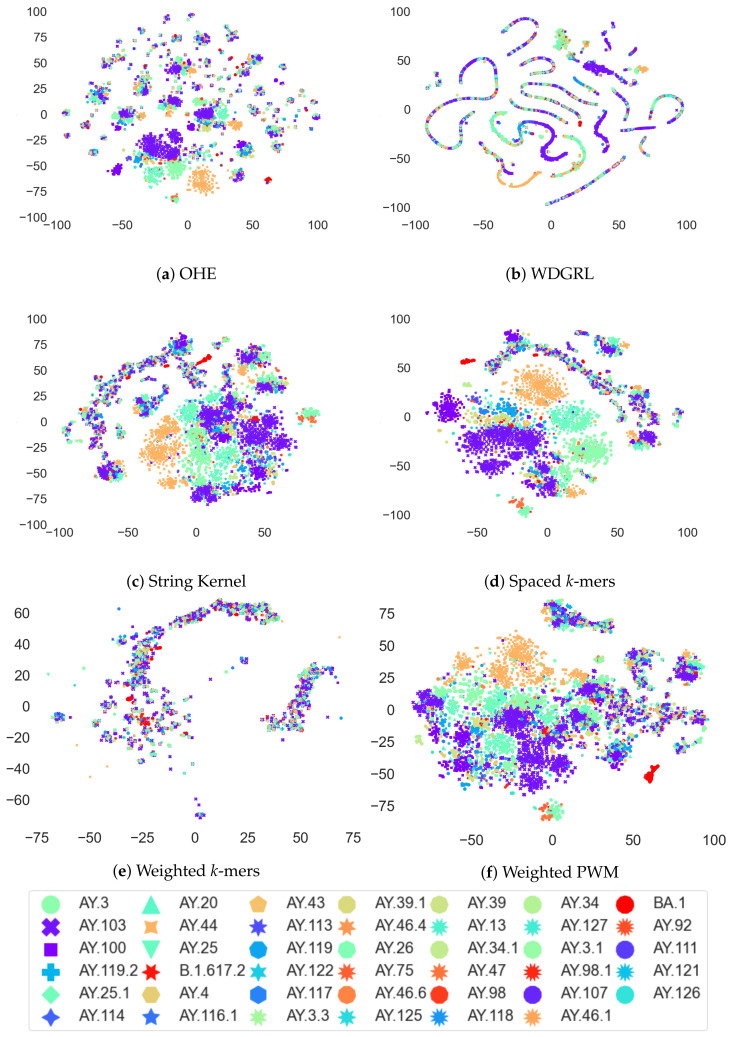
The t-SNE visualizations of the original set of 8172 error-free SARS-CoV-2 sequences employing different embedding techniques, including OHE, WDGRL, string kernel, spaced *k*-mers, weighted *k*-mers, and weighted PWM. The visualizations offer a comparative analysis of the effectiveness of each embedding method, with notable prominence observed for AY.44 variants in the t-SNE plot based on the string kernel vector. For optimal viewing experience, it is advised to refer to the figure in color.

The t-distributed stochastic neighbor embedding (t-SNE) method is a widely used data visualization technique that preserves the pairwise distances between high-dimensional vectors in a lower-dimensional space. In this study, we employed t-SNE to visualize the clustering patterns of different coronavirus variants using various embedding vector generation methods, including one-hot encoding (OHE), Wasserstein-distance-guided representation learning (WDGRL), string kernel, spaced *k*-mer, weighted *k*-mer, and weighted position weight matrix (PWM). Our analysis, as depicted in Figure 6, reveals the remarkable effectiveness of t-SNE in capturing the pairwise distance information and unveiling the distinct grouping patterns of coronavirus variants in a two-dimensional space. Specifically, the t-SNE plot based on the OHE vector demonstrated that AY.44 variants were more clearly grouped than the other variants, while the WDGRL vector maintained a smaller group of variants than OHE vector. Furthermore, the string-kernel-vector-based t-SNE plot exhibited clearer grouping patterns of AY.44 and other variants than the OHE vector. Additionally, the spaced *k*-mer vector method showed a more distinct grouping of variants compared to other embedding vector generation methods. The weighted *k*-mer vector exhibited grouping of the variants similar to the WDGRL vector, whereas the weighted PWM vector showed grouping patterns more similar to the string kernel vector.

## 4. Conclusions

In summary, the COVID-19 pandemic has emphasized the importance of transitioning from second-generation to third-generation sequencing technology. Long-read sequencing has emerged as a critical tool for unraveling various genomic features of the SARS-CoV-2 virus. With the ability to read longer DNA fragments, ranging from 5000 to 30,000 base pairs, long-read sequencing addresses a major challenge faced by short-read sequencing methods. This extended read length has enabled researchers to detect complex structural variations, including large insertions/deletions, inversions, repeats, duplications, and translocations. Additionally, long-read sequencing has facilitated the phasing of SNPs into haplotypes and facilitated de novo genome assembly. However, it is important to acknowledge that the high error rate associated with long-read sequencing may impact the interpretation of SARS-CoV-2’s biology.

In this study, we have demonstrated that the accuracy of machine learning classification algorithms in analyzing SARS-CoV-2 genome sequences greatly depends on the selection of appropriate embedding methods. Our analysis of simulated SARS-CoV-2 viral sequences underscores the value of employing robust embedding techniques capable of effectively managing errors and accurately categorizing genome sequences considering both long-read sequencer-specific errors and random error types. Specifically, we have identified certain embedding methods, such as WDGRL and weighted PWM, as superior in detecting errors and classifying sequences. These findings highlight the potential of machine learning in analyzing SARS-CoV-2 genomic data, contributing to a deeper understanding of the virus’s evolution and spread.

In the future, we want to explore more sequence embedding and advanced deep learning methods on SARS-CoV-2 genomic sequences generated at different long-read sequencing depths with third-generation sequence-specific errors. These experiments will help us develop robust models to improve our ability to adapt long-read sequencing technology (PacBio and ONT) to produce error-free SARS-CoV-2 genome sequences to understand and answer critical biological questions.

## Figures and Tables

**Table 1:** Dataset statistics for different lineages in our data. The total number of SARS-CoV-2 genome sequences (and corresponding lineages) was 8172 after preprocessing.

Lineage	No. Sequences	Lineage	No. Sequences
AY.103	2253	AY.121	40
AY.44	1407	AY.75	36
AY.100	715	AY.3.1	30
AY.3	705	AY.3.3	28
AY.25	582	AY.107	27
AY.25.1	381	AY.34.1	25
AY.39	247	AY.46.6	21
AY.119	241	AY.98.1	20
B.1.617.2	173	AY.13	19
AY.20	129	AY.116.1	18
AY.26	107	AY.126	17
AY.4	99	AY.114	15
AY.117	93	AY.46.1	14
AY.113	92	AY.34	14
AY.118	86	AY.125	14
AY.43	85	AY.92	13
AY.122	84	AY.46.4	12
BA.1	79	AY.98	12
AY.119.2	74	AY.127	12
AY.47	73	AY.111	10
AY.39.1	70	_	_

**Table 2:** Accuracy results obtained from an error-free set of 8172 SARS-CoV-2 genome sequences. The optimal values are highlighted in bold for ease of interpretation.

Embed. Method	ML Algo.	Acc.	Prec.	Recall	F1 Weigh.	F1 Macro	ROC-AUC	Train. Runtime (s)
OHE	SVM	0.773	0.772	0.773	0.766	0.571	0.760	19,231.462
	NB	0.086	0.192	0.086	0.091	0.194	0.595	615.813
	MLP	0.360	0.344	0.360	0.252	0.043	0.514	1222.237
	KNN	0.669	0.689	0.669	0.649	0.409	0.666	38.431
	RF	0.774	0.774	0.774	0.765	0.574	0.758	224.910
	LR	0.721	0.741	0.721	0.707	0.555	0.741	37,539.362
	DT	0.844	0.845	0.844	0.842	0.610	0.796	219.236
WDGRL	SVM	0.327	0.159	0.327	0.203	0.036	0.511	2.789
	NB	0.166	0.169	0.166	0.167	0.018	0.510	0.028
	MLP	0.413	0.318	0.413	0.327	0.077	0.530	21.971
	KNN	0.463	0.431	0.463	0.437	0.192	0.581	0.118
	RF	0.449	0.446	0.449	0.445	0.207	0.601	1.671
	LR	0.323	0.261	0.323	0.201	0.036	0.510	0.752
	DT	0.440	0.441	0.440	0.438	0.195	0.596	0.028
String Kernel	SVM	0.881	0.880	0.881	0.878	0.776	0.880	7.200
	NB	0.033	0.309	0.033	0.032	0.048	0.531	0.556
	MLP	0.715	0.700	0.715	0.704	0.369	0.690	34.899
	KNN	0.749	0.757	0.749	0.735	0.544	0.738	0.648
	RF	0.672	0.750	0.672	0.634	0.395	0.652	10.258
	LR	0.864	0.858	0.864	0.854	0.675	0.817	50.100
	DT	0.572	0.578	0.572	0.573	0.337	0.669	3.636
Spaced *k*-mers	SVM	**0.956**	**0.956**	**0.956**	**0.955**	**0.890**	**0.933**	**8.761**
	NB	0.068	0.305	0.068	0.059	0.184	0.606	0.553
	MLP	0.825	0.832	0.825	0.826	0.539	0.771	14.855
	KNN	0.796	0.808	0.796	0.784	0.611	0.776	0.754
	RF	0.915	0.920	0.915	0.908	0.749	0.835	2.107
	LR	0.956	0.956	0.956	0.954	0.881	0.921	19.964
	DT	0.834	0.836	0.834	0.832	0.647	0.816	0.739
Weighted *k*-mers	SVM	0.293	0.201	0.293	0.158	0.037	0.510	30.304
	NB	0.017	0.014	0.017	0.014	0.027	0.523	0.088
	MLP	0.275	0.168	0.275	0.159	0.037	0.511	16.368
	KNN	0.193	0.186	0.193	0.159	0.052	0.516	0.661
	RF	0.278	0.198	0.278	0.180	0.061	0.519	1.066
	LR	0.285	0.178	0.285	0.164	0.043	0.513	1.648
	DT	0.265	0.174	0.265	0.174	0.055	0.517	0.064
Weighted PWM	SVM	0.852	0.850	0.852	0.849	0.741	0.863	3.257
	NB	0.028	0.015	0.028	0.018	0.048	0.553	0.093
	MLP	0.760	0.740	0.760	0.748	0.393	0.695	28.944
	KNN	0.751	0.755	0.751	0.738	0.555	0.743	0.635
	RF	0.801	0.830	0.801	0.783	0.622	0.759	3.285
	LR	0.861	0.859	0.861	0.856	0.755	0.882	8.702
	DT	0.651	0.662	0.651	0.653	0.391	0.706	0.450

**Table 3:** Provides a comprehensive analysis of the robustness of 8172 SARS-CoV-2 genome sequences under two different sequencing depths (5 and 10) and specific errors associated with the PacBio sequencer. The results of this analysis, which are based on the identification of optimal values, have been highlighted in bold for ease of interpretation.

Embed. Method	ML Algo.	Depth-5 Simulated Error							Depth-10 Simulated Error						
		Acc.	Prec.	Recall	F1 Weigh.	F1 Macro	ROC-AUC	Train. Runtime (s)	Acc.	Prec.	Recall	F1 Weigh.	F1 Macro	ROC- AUC	Train. Runtime (s)
OHE	SVM	0.276	0.076	0.276	0.119	0.011	0.500	181,443.9	0.276	0.076	0.276	0.119	0.011	0.500	188,143.3
	NB	0.276	0.076	0.276	0.119	0.011	0.500	1452.37	0.276	0.076	0.276	0.119	0.011	0.500	1450.059
	MLP	0.087	0.008	0.087	0.014	0.004	0.500	2833.82	0.172	0.030	0.172	0.051	0.007	0.500	1763.060
	KNN	0.276	0.076	0.276	0.119	0.011	0.500	126.632	0.276	0.076	0.276	0.119	0.011	0.500	119.041
	RF	0.172	0.030	0.172	0.051	0.007	0.500	300.639	0.276	0.220	0.276	0.120	0.011	0.500	306.980
	LR	0.021	0.000	0.021	0.001	0.001	0.500	77099.6	0.021	0.000	0.021	0.001	0.001	0.500	71666.1
	DT	0.172	0.030	0.172	0.051	0.007	0.500	890.873	0.030	0.087	0.030	0.002	0.001	0.500	441.523
WDGRL	SVM	0.276	0.076	0.276	0.119	0.011	0.500	8.056	0.276	0.076	0.276	0.119	0.011	0.500	8.117
	NB	0.001	0.000	0.001	0.000	0.000	0.500	0.053	0.001	0.000	0.001	0.000	0.000	0.500	0.052
	MLP	0.276	0.076	0.276	0.119	0.011	0.500	9.374	0.276	0.076	0.276	0.119	0.011	0.500	16.305
	KNN	0.172	0.030	0.172	0.051	0.007	0.500	1.724	0.172	0.030	0.172	0.051	0.007	0.500	1.936
	RF	0.276	0.076	0.276	0.119	0.011	0.500	0.711	0.276	0.076	0.276	0.119	0.011	0.500	0.552
	LR	0.172	0.030	0.172	0.051	0.007	0.500	0.500	0.172	0.030	0.172	0.051	0.007	0.500	0.574
	DT	0.276	0.076	0.276	0.119	0.011	0.500	0.006	0.276	0.076	0.276	0.119	0.011	0.500	0.006
String Kernel	SVM	0.276	0.076	0.276	0.119	0.011	0.500	19.994	0.276	0.076	0.276	0.119	0.011	0.500	18.068
	NB	0.001	0.000	0.001	0.000	0.000	0.500	1.873	0.001	0.000	0.001	0.000	0.000	0.500	1.957
	MLP	0.276	0.076	0.276	0.119	0.011	0.500	71.414	0.276	0.076	0.276	0.119	0.011	0.500	59.946
	KNN	0.276	0.076	0.276	0.119	0.011	0.500	2.390	0.276	0.076	0.276	0.119	0.011	0.500	2.409
	RF	0.276	0.076	0.276	0.119	0.011	0.500	14.867	0.276	0.076	0.276	0.119	0.011	0.500	16.127
	LR	0.276	0.076	0.276	0.119	0.011	0.500	75.829	0.276	0.076	0.276	0.119	0.011	0.500	75.556
	DT	0.087	0.008	0.087	0.014	0.004	0.500	6.013	0.029	0.001	0.029	0.002	0.001	0.500	5.979
**Spaced *k*-mers**	**SVM**	**0.276**	**0.076**	**0.276**	**0.119**	**0.011**	**0.500**	**73.149**	**0.276**	**0.076**	**0.276**	**0.119**	**0.011**	**0.500**	**60.317**
	**NB**	**0.276**	**0.076**	**0.276**	**0.119**	**0.011**	**0.500**	**6.302**	**0.276**	**0.076**	**0.276**	**0.119**	**0.011**	**0.500**	**4.487**
	**MLP**	**0.276**	**0.076**	**0.276**	**0.119**	**0.011**	**0.500**	**126.596**	0.016	0.000	0.016	0.000	0.001	0.500	101.059
	**KNN**	**0.276**	**0.076**	**0.276**	**0.119**	**0.011**	**0.500**	**1.945**	**0.276**	**0.076**	**0.276**	**0.119**	**0.011**	**0.500**	**2.076**
	**RF**	**0.276**	**0.076**	**0.276**	**0.119**	**0.011**	**0.500**	**8.278**	**0.276**	**0.076**	**0.276**	**0.119**	**0.011**	**0.500**	**3.487**
	**LR**	**0.276**	**0.076**	**0.276**	**0.119**	**0.011**	**0.500**	**23.153**	**0.276**	**0.076**	**0.276**	**0.119**	**0.011**	**0.500**	**19.397**
	DT	0.086	0.007	0.086	0.014	0.004	0.500	1.455	0.172	0.030	0.172	0.051	0.007	0.500	0.433
Weighted *k*-mers	SVM	0.276	0.076	0.276	0.120	0.011	0.500	63.260	0.276	0.076	0.276	0.120	0.011	0.500	62.970
	NB	0.001	0.000	0.001	0.000	0.000	0.500	0.870	0.001	0.000	0.001	0.000	0.000	0.500	0.697
	MLP	0.276	0.076	0.276	0.120	0.011	0.500	29.469	0.276	0.076	0.276	0.120	0.011	0.500	25.876
	KNN	0.071	0.005	0.071	0.009	0.003	0.500	1.970	0.071	0.005	0.071	0.009	0.003	0.500	1.979
	RF	0.276	0.076	0.276	0.120	0.011	0.500	1.683	0.276	0.076	0.276	0.120	0.011	0.500	1.640
	LR	0.276	0.076	0.276	0.120	0.011	0.500	2.404	0.276	0.076	0.276	0.120	0.011	0.500	2.378
	DT	0.276	0.076	0.276	0.120	0.011	0.500	0.098	0.276	0.076	0.276	0.120	0.011	0.500	0.097
Weighted PWM	SVM	0.276	0.079	0.276	0.120	0.013	0.501	8.651	0.276	0.076	0.276	0.120	0.011	0.500	10.205
	NB	0.002	0.000	0.002	0.000	0.000	0.500	0.787	0.002	0.000	0.002	0.000	0.000	0.500	0.622
	MLP	0.005	0.000	0.005	0.000	0.000	0.500	24.250	0.005	0.000	0.005	0.000	0.000	0.500	31.357
	KNN	0.276	0.076	0.276	0.120	0.011	0.500	2.358	0.276	0.076	0.276	0.120	0.011	0.500	2.828
	RF	0.172	0.030	0.172	0.051	0.007	0.500	4.826	0.276	0.076	0.276	0.120	0.011	0.500	6.342
	LR	0.013	0.000	0.013	0.000	0.001	0.500	13.294	0.013	0.000	0.013	0.000	0.001	0.500	17.801
	DT	0.071	0.005	0.071	0.009	0.003	0.500	0.694	0.046	0.002	0.046	0.004	0.002	0.500	0.793

**Table 4:** Provides a comprehensive analysis of the robustness of 8172 SARS-CoV-2 genome sequences under two different sequencing depths (5 and 10) and specific errors associated with the Oxford Nanopore Technology (ONT) sequencer. The results of this analysis, which are based on the identification of optimal values, have been highlighted in bold for ease of interpretation.

Embed. Method	ML Algo.	n2020 5× Simulated Error							n2020 10× Simulated Error						
		Acc.	Prec.	Recall	F1 Weigh.	F1 Macro	ROC-AUC	Train. Runtime (s)	Acc.	Prec.	Recall	F1 Weigh.	F1 Macro	ROC-AUC	Train. Runtime (s)
OHE	SVM	0.122	0.133	0.122	0.112	0.022	0.501	70,679.0	0.106	0.127	0.106	0.094	0.018	0.500	103,909.2
	NB	0.276	0.076	0.276	0.119	0.011	0.500	1075.20	0.276	0.076	0.276	0.119	0.011	0.500	833.594
	MLP	0.071	0.097	0.071	0.010	0.003	0.500	3906.45	0.276	0.076	0.276	0.119	0.011	0.500	1539.707
	KNN	0.189	0.130	0.189	0.134	0.019	0.501	82.195	0.189	0.133	0.189	0.134	0.017	0.500	108.649
	RF	0.072	0.141	0.072	0.076	0.008	0.500	276.773	0.241	0.116	0.241	0.147	0.016	0.500	319.020
	LR	0.272	0.108	0.272	0.122	0.011	0.500	67103.9	0.270	0.130	0.270	0.124	0.012	0.500	68286.4
	DT	0.166	0.134	0.166	0.103	0.017	0.501	460.552	0.163	0.127	0.163	0.087	0.013	0.500	411.342
WDGRL	SVM	0.244	0.108	0.244	0.147	0.015	0.501	8.906	0.214	0.119	0.214	0.138	0.016	0.502	8.812
	NB	0.011	0.094	0.011	0.019	0.002	0.496	0.063	0.184	0.129	0.184	0.089	0.011	0.501	0.060
	MLP	0.268	0.085	0.268	0.122	0.012	0.500	30.652	0.192	0.137	0.192	0.116	0.013	0.502	28.833
	KNN	0.261	0.150	0.261	0.127	0.013	0.500	0.382	0.185	0.145	0.185	0.120	0.018	0.502	0.374
	RF	0.109	0.147	0.109	0.080	0.012	0.499	2.572	0.153	0.151	0.153	0.090	0.014	0.500	2.383
	LR	0.125	0.073	0.125	0.074	0.009	0.500	1.088	0.191	0.167	0.191	0.088	0.011	0.501	1.058
	DT	0.103	0.148	0.103	0.074	0.012	0.499	0.048	0.166	0.157	0.166	0.093	0.014	0.501	0.047
String Kernel	SVM	0.144	0.134	0.144	0.137	0.024	0.500	20.179	0.154	0.142	0.154	0.147	0.025	0.501	18.168
	NB	0.004	0.000	0.004	0.000	0.001	0.503	2.019	0.004	0.000	0.004	0.000	0.001	0.499	1.803
	MLP	0.137	0.135	0.137	0.135	0.026	0.501	59.144	0.132	0.139	0.132	0.134	0.028	0.502	49.297
	KNN	0.195	0.123	0.195	0.132	0.020	0.500	2.877	0.189	0.137	0.189	0.147	0.022	0.500	2.807
	RF	0.249	0.116	0.249	0.144	0.015	0.500	15.951	0.263	0.157	0.263	0.153	0.016	0.501	15.812
	LR	0.157	0.130	0.157	0.141	0.023	0.500	78.382	0.171	0.140	0.171	0.153	0.024	0.500	76.744
	DT	0.130	0.129	0.130	0.129	0.023	0.500	5.910	0.137	0.140	0.137	0.137	0.026	0.501	5.969
Spaced *k*-mers	SVM	0.132	0.147	0.132	0.133	0.039	0.508	23.890	0.193	0.208	0.193	0.198	0.092	0.535	20.470
	NB	0.004	0.000	0.004	0.000	0.002	0.503	3.843	0.006	0.003	0.006	0.001	0.004	0.502	4.476
	MLP	0.112	0.142	0.112	0.118	0.025	0.502	58.597	0.176	0.192	0.176	0.178	0.051	0.514	44.872
	KNN	0.225	0.150	0.225	0.146	0.018	0.500	3.024	0.222	0.177	0.222	0.181	0.035	0.508	2.920
	RF	0.206	0.148	0.206	0.162	0.033	0.506	4.026	0.232	0.196	0.232	0.207	0.062	0.519	4.038
	LR	0.135	0.151	0.135	0.137	0.040	0.509	70.516	0.198	0.211	0.198	0.202	0.097	0.538	57.721
	DT	0.131	0.140	0.131	0.128	0.028	0.502	1.050	0.145	0.176	0.145	0.157	0.050	0.516	1.011
**Weighted *k*-mers**	**SVM**	**0.276**	**0.076**	**0.276**	**0.119**	**0.011**	**0.500**	**73.858**	**0.276**	**0.076**	**0.276**	**0.119**	**0.011**	**0.500**	**74.108**
	NB	0.001	0.000	0.001	0.000	0.000	0.500	0.778	0.001	0.000	0.001	0.000	0.000	0.500	0.805
	**MLP**	**0.276**	**0.076**	**0.276**	**0.119**	**0.011**	**0.500**	**30.145**	**0.276**	**0.076**	**0.276**	**0.119**	**0.011**	**0.500**	**31.810**
	KNN	0.172	0.030	0.172	0.051	0.007	0.500	2.079	0.172	0.030	0.172	0.051	0.007	0.500	1.937
	**RF**	**0.276**	**0.076**	**0.276**	**0.119**	**0.011**	**0.500**	**1.763**	**0.276**	**0.076**	**0.276**	**0.119**	**0.011**	**0.500**	**1.645**
	**LR**	**0.276**	**0.076**	**0.276**	**0.119**	**0.011**	**0.500**	**2.557**	**0.276**	**0.076**	**0.276**	**0.119**	**0.011**	**0.500**	**2.473**
	**DT**	**0.276**	**0.076**	**0.276**	**0.119**	**0.011**	**0.500**	**0.116**	**0.276**	**0.076**	**0.276**	**0.119**	**0.011**	**0.500**	**0.116**
Weighted PWM	SVM	0.203	0.141	0.203	0.157	0.023	0.501	11.123	0.240	0.170	0.240	0.177	0.028	0.504	9.825
	NB	0.002	0.000	0.002	0.000	0.000	0.498	0.808	0.003	0.000	0.003	0.000	0.000	0.500	0.814
	MLP	0.086	0.141	0.086	0.091	0.022	0.501	36.325	0.119	0.180	0.119	0.132	0.035	0.513	27.304
	KNN	0.193	0.127	0.193	0.129	0.017	0.501	2.745	0.188	0.148	0.188	0.151	0.025	0.503	2.475
	RF	0.175	0.141	0.175	0.151	0.031	0.505	5.887	0.203	0.175	0.203	0.181	0.042	0.510	4.699
	LR	0.084	0.152	0.084	0.093	0.026	0.505	16.420	0.143	0.194	0.143	0.155	0.051	0.519	12.654
	DT	0.069	0.128	0.069	0.077	0.021	0.501	0.830	0.101	0.152	0.101	0.115	0.030	0.504	0.670

**Table 5:** Provides a comprehensive analysis of the robustness of 8172 SARS-CoV-2 genome sequences under two different sequencing depths (5 and 10) and incorporated with random errors. The results of this analysis, which are based on the identification of optimal values, have been highlighted in bold for ease of interpretation.

Embed. Method	ML Algo.	Random 5× Simulated Error							Random 10× Simulated Error						
		Acc.	Prec.	Recall	F1 Weigh.	F1 Macro	ROC-AUC	Train. Runtime (s)	Acc.	Prec.	Recall	F1 Weigh.	F1 Macro	ROC-AUC	Train. Runtime (s)
OHE	SVM	0.101	0.138	0.101	0.078	0.016	0.500	157054.2	0.107	0.145	0.107	0.093	0.019	0.501	74,697.7
	NB	0.276	0.076	0.276	0.119	0.011	0.500	1113.271	0.276	0.076	0.276	0.119	0.011	0.500	1119.210
	MLP	0.013	0.000	0.013	0.000	0.002	0.500	1503.268	0.267	0.085	0.267	0.125	0.012	0.500	1625.962
	KNN	0.223	0.131	0.223	0.136	0.018	0.500	91.708	0.204	0.135	0.204	0.139	0.018	0.501	100.443
	RF	0.202	0.112	0.202	0.126	0.014	0.500	259.562	0.186	0.114	0.186	0.112	0.013	0.500	405.555
	LR	0.271	0.109	0.271	0.122	0.011	0.500	70035.07	0.272	0.123	0.272	0.122	0.012	0.500	83434.3
	DT	0.157	0.126	0.157	0.075	0.012	0.500	776.263	0.164	0.136	0.164	0.086	0.014	0.500	943.475
WDGRL	SVM	0.276	0.076	0.276	0.119	0.011	0.500	8.776	0.275	0.118	0.275	0.158	0.017	0.502	8.842
	NB	0.001	0.000	0.001	0.000	0.000	0.500	0.068	0.005	0.105	0.005	0.007	0.001	0.500	0.051
	MLP	0.276	0.076	0.276	0.119	0.011	0.500	31.487	0.272	0.129	0.272	0.125	0.012	0.500	21.765
	KNN	0.276	0.076	0.276	0.119	0.011	0.500	0.348	0.169	0.142	0.169	0.119	0.016	0.500	0.383
	RF	0.087	0.008	0.087	0.014	0.004	0.500	2.488	0.072	0.138	0.072	0.086	0.015	0.499	2.510
	LR	0.087	0.008	0.087	0.014	0.004	0.500	1.069	0.267	0.115	0.267	0.127	0.012	0.500	1.059
	DT	0.001	0.000	0.001	0.000	0.000	0.500	0.046	0.068	0.135	0.068	0.083	0.015	0.499	0.046
String Kernel	SVM	0.169	0.132	0.169	0.142	0.024	0.501	19.308	0.140	0.128	0.140	0.133	0.023	0.500	18.115
	NB	0.003	0.004	0.003	0.001	0.002	0.501	1.894	0.004	0.000	0.004	0.000	0.001	0.500	1.811
	MLP	0.138	0.135	0.138	0.134	0.023	0.500	83.035	0.124	0.130	0.124	0.127	0.024	0.500	60.808
	KNN	0.213	0.128	0.213	0.130	0.017	0.500	2.773	0.211	0.136	0.211	0.142	0.020	0.501	2.816
	RF	0.263	0.140	0.263	0.140	0.014	0.500	15.649	0.257	0.122	0.257	0.146	0.015	0.500	15.715
	LR	0.180	0.132	0.180	0.148	0.022	0.500	79.243	0.158	0.129	0.158	0.141	0.022	0.500	76.412
	DT	0.134	0.129	0.134	0.129	0.023	0.500	6.318	0.125	0.130	0.125	0.127	0.022	0.499	5.773
Spaced *k*-mers	SVM	0.080	0.139	0.080	0.080	0.012	0.501	17.010	0.124	0.155	0.124	0.118	0.022	0.501	22.282
	NB	0.005	0.004	0.005	0.001	0.002	0.502	3.879	0.006	0.000	0.006	0.001	0.002	0.500	4.803
	MLP	0.133	0.111	0.133	0.060	0.011	0.500	41.950	0.116	0.163	0.116	0.107	0.019	0.502	47.611
	KNN	0.263	0.092	0.263	0.121	0.012	0.500	2.763	0.246	0.143	0.246	0.125	0.014	0.500	2.646
	RF	0.210	0.128	0.210	0.148	0.019	0.501	3.921	0.233	0.139	0.233	0.159	0.023	0.502	3.587
	LR	0.147	0.144	0.147	0.116	0.018	0.501	64.852	0.135	0.171	0.135	0.128	0.027	0.505	60.750
	DT	0.156	0.128	0.156	0.088	0.015	0.501	0.952	0.127	0.135	0.127	0.096	0.020	0.501	1.006
**Weighted *k*-mers**	**SVM**	**0.276**	**0.076**	**0.276**	**0.119**	**0.011**	**0.500**	**76.220**	**0.276**	**0.076**	**0.276**	**0.119**	**0.011**	**0.500**	**78.497**
	NB	0.001	0.000	0.001	0.000	0.000	0.500	0.774	0.001	0.000	0.001	0.000	0.000	0.500	0.749
	**MLP**	**0.276**	**0.076**	**0.276**	**0.119**	**0.011**	**0.500**	**23.639**	**0.276**	**0.076**	**0.276**	**0.119**	**0.011**	**0.500**	**34.219**
	KNN	0.172	0.030	0.172	0.051	0.007	0.500	1.932	0.172	0.030	0.172	0.051	0.007	0.500	1.915
	**RF**	**0.276**	**0.076**	**0.276**	**0.119**	**0.011**	**0.500**	**1.851**	**0.276**	**0.076**	**0.276**	**0.119**	**0.011**	**0.500**	**1.749**
	**LR**	**0.276**	**0.076**	**0.276**	**0.119**	**0.011**	**0.500**	**2.706**	**0.276**	**0.076**	**0.276**	**0.119**	**0.011**	**0.500**	**2.626**
	**DT**	**0.276**	**0.076**	**0.276**	**0.119**	**0.011**	**0.500**	**0.100**	**0.276**	**0.076**	**0.276**	**0.119**	**0.011**	**0.500**	**0.102**
Weighted PWM	SVM	0.203	0.133	0.203	0.145	0.020	0.500	11.330	0.196	0.143	0.196	0.151	0.024	0.501	8.605
	NB	0.002	0.000	0.002	0.000	0.000	0.500	0.594	0.002	0.000	0.002	0.000	0.000	0.500	0.766
	MLP	0.016	0.087	0.016	0.015	0.004	0.499	43.680	0.037	0.110	0.037	0.036	0.012	0.501	29.312
	KNN	0.214	0.108	0.214	0.122	0.015	0.500	2.758	0.201	0.143	0.201	0.130	0.017	0.500	2.618
	RF	0.163	0.122	0.163	0.129	0.017	0.501	5.649	0.181	0.134	0.181	0.136	0.019	0.501	4.915
	LR	0.061	0.104	0.061	0.058	0.010	0.499	16.697	0.070	0.131	0.070	0.074	0.014	0.502	12.835
	DT	0.061	0.138	0.061	0.058	0.015	0.500	0.875	0.073	0.136	0.073	0.070	0.018	0.500	0.673

## Data Availability

The data utilized in this research study were acquired from the publicly accessible database known as the Global Initiative on Sharing All Influenza Data (GISAID) (https://www.gisaid.org/ (accessed on 19 May 2023)) for the period spanning September to December 2021. To facilitate replication of the findings, the source codes and pipelines employed in the analysis can be accessed at https://github.com/sarwanpasha/Long_Read_Noisy_Sequences (accessed on 19 May 2023).

## Abbreviations

The following abbreviations are used in this manuscript:

WGS	Whole Genome Sequencing
TGS	Third-Generation Sequencing
GISAID	Global Initiative on Sharing All Influenza Data
ML	Machine Learning
DL	Deep Learning
WDGRL	Wasserstein Distance-based Generative Adversarial Network for Representation Learning
PacBio	Pacific Biosciences
ONT	Oxford Nanopore Technologies
OHE	One-Hot Encoding
PWM	Position Weight Matrix
SVM	Support Vector Machine
NB	Naïve Bayes
MLP	Multi-Layer Perceptron
KNN	*K*-Nearest Neighbors
RF	Random Forest
LR	Logistic Regression
DT	Decision Tree

## References

[1] Wu F., Zhao S., Yu B., Chen Y.M., Wang W., Song Z.G., Hu Y., Tao Z.W., Tian J.H., Pei Y.Y. (2020). A new coronavirus associated with human respiratory disease in China. Nature.

[2] Kim D., Lee J.Y., Yang J.S., Kim J.W., Kim V.N., Chang H. (2020). The Architecture of SARS-CoV-2 Transcriptome. Cell.

[3] Park S.E. (2020). Epidemiology, virology, and clinical features of severe acute respiratory syndrome -coronavirus-2 (SARS-CoV-2; Coronavirus Disease-19). Clin. Exp. Pediatr..

[4] Rambaut A., Holmes E.C., O’Toole Á., Hill V., McCrone J.T., Ruis C., du Plessis L., Pybus O.G. (2020). A dynamic nomenclature proposal for SARS-CoV-2 lineages to assist genomic epidemiology. Nat. Microbiol..

[5] (2021). GISAID Website. https://www.gisaid.org/.

[6] Hadfield J., Megill C., Bell S.M., Huddleston J., Potter B., Callender C., Sagulenko P., Bedford T., Neher R.A. (2018). Nextstrain: Real-time tracking of pathogen evolution. Bioinformatics.

[7] Aksamentov I., Roemer C., Hodcroft E., Neher R. (2021). Nextclade: Clade assignment, mutation calling and quality control for viral genomes. J. Open Source Softw..

[8] Gardy J.L., Loman N.J. (2018). Towards a genomics-informed, real-time, global pathogen surveillance system. Nat. Reviews. Genet..

[9] Arons M.M., Hatfield K.M., Reddy S.C., Kimball A., James A., Jacobs J.R., Taylor J., Spicer K., Bardossy A.C., Oakley L.P. (2020). Presymptomatic SARS-CoV-2 Infections and Transmission in a Skilled Nursing Facility. N. Engl. J. Med..

[10] Korber B., Fischer W., Gnanakaran S. (2020). Tracking changes in SARS-CoV-2 Spike: Evidence that D614G increases infectivity of the COVID-19 virus. Cell.

[11] Rhoads A., Au K.F. (2015). PacBio Sequencing and Its Applications. Genom. Proteom. Bioinform..

[12] Laver T., Harrison J., O’Neill P., Moore K., Farbos A., Paszkiewicz K., Studholme D. (2015). Assessing the performance of the Oxford Nanopore Technologies MinION. Biomol. Detect. Quantif..

[13] McCarthy A. (2010). Third Generation DNA Sequencing: Pacific Biosciences’ Single Molecule Real Time Technology. Chem. Biol..

[14] Lu H., Giordano F., Ning Z. (2016). Oxford Nanopore MinION Sequencing and Genome Assembly. Genom. Proteom. Bioinform..

[15] Reuter J., Spacek D.V., Snyder M. (2015). High-Throughput Sequencing Technologies. Mol. Cell.

[16] Singh O.P., Vallejo M., El-Badawy I.M., Aysha A., Madhanagopal J., Mohd Faudzi A.A. (2021). Classification of SARS-CoV-2 and non-SARS-CoV-2 using machine learning algorithms. Comput. Biol. Med..

[17] Ali S., Sahoo B., Ullah N., Zelikovskiy A., Patterson M., Khan I. A k-mer based approach for sars-cov-2 variant identification. Proceedings of the International Symposium on Bioinformatics Research and Applications.

[18] Kuzmin K., Adeniyi A.E., DaSouza A.K., Lim D., Nguyen H., Molina N.R., Xiong L., Weber I.T., Harrison R.W. (2020). Machine learning methods accurately predict host specificity of coronaviruses based on spike sequences alone. Biochem. Biophys. Res. Commun..

[19] Singh R., Sekhon A., Kowsari K., Lanchantin J., Wang B., Qi Y. Gakco: A fast gapped k-mer string kernel using counting. Proceedings of the Joint European Conference on Machine Learning and Knowledge Discovery in Databases.

[20] Ali S., Sahoo B., Zelikovsky A., Chen P.Y., Patterson M. (2023). Benchmarking machine learning robustness in COVID-19 genome sequence classification. Sci. Rep..

[21] Ono Y., Asai K., Hamada M. (2012). PBSIM: PacBio reads simulator—toward accurate genome assembly. Bioinformatics.

[22] Li H. (2018). Minimap2: Pairwise alignment for nucleotide sequences. Bioinformatics.

[23] Danecek P., Bonfield J.K., Liddle J., Marshall J., Ohan V., Pollard M.O., Whitwham A., Keane T., McCarthy S.A., Davies R.M. (2021). Twelve years of SAMtools and BCFtools. GigaScience.

[24] Wick R. (2019). Badread: Simulation of error-prone long reads. J. Open Source Softw..

[25] Shen J., Qu Y., Zhang W., Yu Y. Wasserstein distance guided representation learning for domain adaptation. Proceedings of the AAAI Conference on Artificial Intelligence.

[26] Farhan M., Tariq J., Zaman A., Shabbir M., Khan I. Efficient Approximation Algorithms for Strings Kernel Based Sequence Classification. Proceedings of the Advances in Neural Information Processing Systems (NeurIPS).

[27] Ali S., Bello B., Chourasia P., Punathil R.T., Zhou Y., Patterson M. (2022). PWM2Vec: An Efficient Embedding Approach for Viral Host Specification from Coronavirus Spike Sequences. Biology.

[28] Van Der Maaten L. (2014). Accelerating t-SNE using tree-based algorithms. J. Mach. Learn. Res..

